# Hepatobiliary manifestations of ulcerative colitis: an example of gut–liver crosstalk

**DOI:** 10.1093/gastro/gou036

**Published:** 2014-06-20

**Authors:** Udayakumar Navaneethan

**Affiliations:** Department of Gastroenterology/Hepatology, Digestive Disease Institute, The Cleveland Clinic Foundation, Cleveland, Ohio, USA

**Keywords:** primary sclerosing cholangitis, restorative proctocolectomy, ulcerative colitis, liver transplantation

## Abstract

The interaction between inflammatory bowel disease (IBD) and hepatobiliary manifestations represents a classic example of liver–gut crosstalk. The importance of liver–gut crosstalk in IBD is demonstrated in the pathogenesis and outcome of primary sclerosing cholangitis (PSC) in IBD patients. Immunoglobulin G4-associated cholangitis (IAC), which has recently been described in UC patients, may also illustrate the significance of gut–liver interaction in these patients. Presence of these hepatobiliary manifestations influences the outcome of associated IBD, in particular ulcerative colitis (UC), and *vice versa*. The pathogenesis of PSC is postulated to be related to gut inflammation in IBD that results in inflammation in the portal tracts (the ‘leaky gut’). Enterohepatic circulation of lymphocytes from the gut to the liver is also of potential relevance to PSC pathogenesis and outcomes. The presence of PSC and gut inflammation in IBD influences the course and outcomes of both diseases. Further research is required, to understand the mutual effect of liver–gut crosstalk in the outcomes of UC patients, and highlights the importance of an interdisciplinary approach—involving gastroenterologists, hepatologists, advanced endoscopists and liver transplant surgeons—in the management of these patients.

## INTRODUCTION

Inflammatory bowel disease (IBD) is associated with extra-intestinal manifestations (EIM) in a significant proportion of patients [1–4]. Hepatobiliary manifestations constitute one of most common EIMs in IBD [5]. Hepatobiliary manifestations are much more commonly associated with ulcerative colitis (UC) than Crohn’s disease (CD). The pathogenesis of hepatobiliary manifestations associated with UC may be related to liver gut cross talk such as in primary sclerosing cholangitis (PSC), PSC/autoimmune hepatitis (AIH) overlap and IgG4 associated cholangitis (IAC). It may also parallel the pathophysiological changes seen with IBD including cholelithiasis, portal vein thrombosis and hepatic abscess [6].

The main goals of this review article are to highlight the importance of liver–gut crosstalk and its significance in the pathogenesis of various hepatobiliary manifestations—in particular PSC and IAC—and its influence on outcomes.

## PRIMARY SCLEROSING CHOLANGITIS

PSC is a cholestatic hepatobiliary disorder affecting the young and middle-aged, and is commonly seen in patients with underlying IBD [6, 7]. PSC is the most common hepatobiliary manifestation seen in IBD [1]. IBD is present in 70–80% of PSC patients [8]. In patients with PSC and IBD, 85–90% have UC and the remaining have Crohn’s colitis or Crohn’s ileocolitis [6].

PSC remains a challenge because its etiology and pathogenesis are still largely unknown. Since sclerosing cholangitis results from any bile duct injury, it is difficult to elucidate the etiology of PSC.

### Pathogenesis of PSC

The etiology and pathogenesis of PSC are not clear. Multiple genetic factors associated with susceptibility have been reported, including human leukocyte anti-gens (HLA)-B8, HLA-DRB1*0301 (DR3), HLA-DRB3*0101 (DRw52a), and HLA-DRB1*0401 (DR4) [9, 10]. Genetic risk factors for the pathogenesis of PSC were investigated for single-nucleotide polymorphisms (SNPs) genotyped using Immunochip (Illumina inc, San Diego CA) analysis and observed that the most notable abnormality was detected in pathways such as PRKD2, HDAC7, and SIK2 [11, 12].

Bile duct injury in PSC is also suggested to be secondary to bile acid toxicity. Changes in the permeability of bile ducts increase permeation of toxic bile acids [13, 14]; also, alterations in the regulation of enterohepatic circulation of bile acids, including the role of the farnesoid X receptor (FXR) and fibroblast growth factor 19 (FGF19), contribute to development of PSC [15-18].

Variations in host immunity play an important role in the pathogenesis of PSC. Concurrent immune-mediated disorders are common in PSC-IBD patients [19]. A variety of auto-antibodies have been detected with PSC, including anti-nuclear antibodies in 24–53%, smooth muscle antibodies in 13–20%, and anti–perinuclear cytoplasmic anti-body (pANCA) in 65–88%, of the patients [20–23]. Proponents of the auto-immune phenomenon argue that the shared epitope in the colonic and biliary epithelia, the presence of tropomyosin-5 is suggested to explain the link between PSC and IBD contributing to both colonic and liver inflammation [24]; however, the lack of disease-specific auto-antibodies—and the lack of PSC response to conventional immunosuppressive therapy—refute the idea of isolated auto-immunity contributing to the pathogenesis [25, 26].

### Liver–gut crosstalk

The pathogenesis of PSC has been suggested to be related to IBD and inflammation in the portal tracts. This ‘leaky gut’ may be related to gut microbiota and may contribute to the pathogenesis of PSC [26]. One of the recently studied genes, FUT2 in PSC and IBD, illustrates the influence of genes on gut microbiota [27, 28]. Similarly, the influence of saturated fat on changes in the bile acid pool, with increase in taurocholic acid, has been postulated to alter the microbiome favoring UC; a similar phenomenon is also being investigated in PSC [29].

Microbiological studies have also found possible links between PSC and IBD. Bacterial `translocation or absorption of bacterial endotoxins into the portal circulation, through a chronically inflamed bowel with the activation of Kupffer cell, may contribute to the pathogenesis of PSC [30, 31]. This notion was supported by the finding that inoculation of enteric bacteria into the portal vein in a rat model caused liver inflammation similar to PSC [32]. Although no single microbial pathogen has been shown to be specifically associated with PSC, these observations support the pivotal role of bacteria and the host’s abnormal immune response in the pathogenesis of PSC [33].

Another possible liver–gut crosstalk in PSC and UC pathogenesis has been suggested to be related to the enterohepatic circulation of lymphocytes [34]. Gut-activated T-lymphocytes in UC patients may contribute to bile duct inflammation because the adhesion molecule profiles of gut and liver endothelium (mucosal vascular addressin cell adhesion molecule 1 and vascular cell adhesion molecule 1 expression along with chemokine C-C motif ligand 25 secretion) are similar [34, 35]. The fact that expression of Mad-CAM-1 in PSC livers depends on the role played by vascular adhesion protein 1 may suggest that modulation of these proteins could affect PSC progression [36].

### UC in PSC

The presentation and behavior of UC in PSC patients is distinctive. Patients with UC with concomitant PSC may demonstrate a higher prevalence of rectal sparing, backwash ileitis, extensive colitis, colorectal neoplasia, and a poorer overall survival rate than patients without concomitant PSC [37–44]. We have also observed that patients with PSC had more marked endoscopic and histological inflammation of the afferent limb if they had undergone restorative proctocolectomy with ileal pouch-anal anastomosis (IPAA) for UC, than those with no concurrent PSC [45]. We will subsequently discuss the impact of liver–gut crosstalk on disease behavior and outcomes.

### Impact of co-existing PSC (before liver transplantation) on disease behavior and course of UC before colectomy

Primary sclerosing cholangitis-ulcerative colitis (PSC-UC) may represent a variant form with distinct histological features and clinical phenotype [37–44]. The sequence of UC diagnosis and PSC diagnosis may vary; for example, new-onset PSC can be diagnosed years after UC diagnosis, with or without proctocolectomy [42].

**Figure 1.**
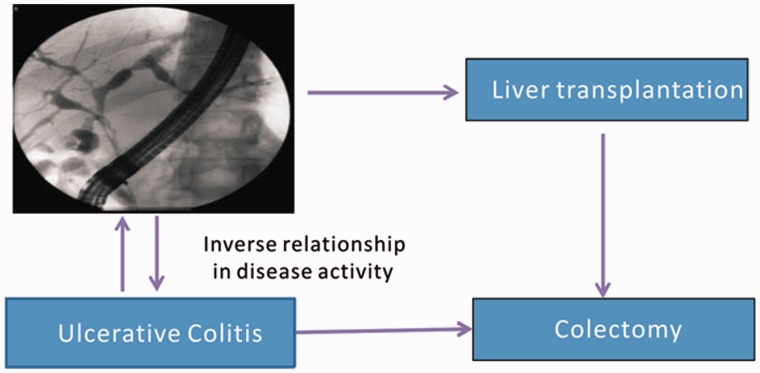
The figure illustrates the inverse relationship between the activity of primary sclerosing cholangitis (PSC) and ulcerative colitis (UC); however, it is important to realize that the severity of PSC does not influence the risk of colon cancer.

The presence of PSC may affect the disease activity of UC before or after colectomy. PSC-UC may more likely run a quiescent course of colitis than in UC patients without co-existent PSC. PSC-UC patients were reported to have a lower grade of histological inflammation of the colon—and a more a prolonged subclinical phase of UC—than patients with UC alone [41, 42]. This observation of sub-clinical activity of UC is only seen in patients who have progressive PSC requiring orthotopic liver transplantation (OLT) [46]. This is hypothesized to be possibly related to depressed T-cell function from cirrhosis and therefore attenuated T-cell-mediated intestinal inflammation [47]. In fact two studies, including ours, have clearly shown an inverse association between UC and PSC disease activity [46, 48]. (Figure 1)

The presence of PSC is associated with an increased risk of UC-associated dysplasia [49]; however the severity of PSC, either mild or severe, does not appear to alter the risk of cancer in UC patients [50]. Given the increased risk of cancer, PSC patients should have screening colonoscopy at the time of PSC diagnosis to evaluate the presence or absence of UC [6]. Also, PSC patients with an established diagnosis of UC or Crohn’s colitis should undergo yearly surveillance colonoscopy. At the time of surveillance colonoscopy, these patients often lack evidence of inflammation; hence random biopsies need to be performed and have been shown to increase the diagnostic yield of dysplasia in patients with PSC and UC [51]. We also observed that, with the implementation of annual colonoscopic surveillance, dysplasia and cancer in patients with a combined diagnosis of PSC-UC is being diagnosed in patients with a shorter duration of these conditions [52]; however, the nature and the location of neoplasia has not changed.

It has also been demonstrated that PSC patients who have elevated IgG4 are more likely to have backwash ileitis, and had reduced colectomy-free survival, compared with patients with normal IgG4 [53]; hence, evaluation of serum IgG4 needs to part of their clinical evaluation.

### Impact of co-existing PSC (after liver transplantation) on disease behavior and course of UC before colectomy

**Figure 2.**
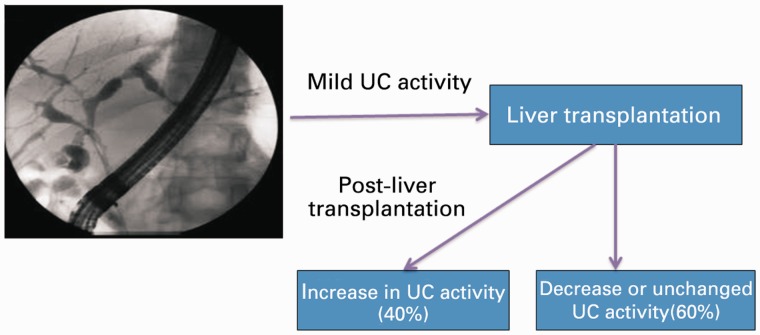
The figure illustrates the relationship between the activity of ulcerative colitis (UC) after liver transplantation. Close to 40% of patients may have worsening of disease activity.

In patients who have undergone OLT for PSC, there is restoration of T-cell function and, theoretically, colonic inflammation should worsen [54]; however, the use of immunosuppressive medications following OLT would contribute to reduction of colon inflammation; hence, the disease activity in IBD patients following OLT is very variable in published studies thus far. Some studies, including ours, have shown that the activity of IBD remains quiescent in most patients after OLT, with only a minority worsening [55, 56], while other studies have shown poor control of IBD in up to 50% of patients following OLT [57, 58]. All these studies are limited by small sample size, lack of uniformity in comparing disease activity and different regimens of immunosuppression following OLT. In one of the largest published studies from Norway, the activity of IBD in 218 PSC patients, before and after OLT, was compared and IBD disease activity either decreased or remained unchanged in 60% of them, while 40% had worsening of their IBD disease activity after OLT (Figure 2) [59]. Younger age at IBD diagnosis and combined treatment with tacrolimus and mycophenolate mofetil (MMF) were associated with increased IBD activity after OLT, whereas combination treatment with cyclosporine and azathioprine was associated with decreased IBD activity following OLT.

The risk of colon cancer in UC patients also persists after OLT; hence, these patients require yearly follow-up surveillance colonoscopy for colon neoplasia, even in patients with no disease activity on colonoscopy [54]. It is not clear whether the risk of colon neoplasia is increased following OLT as a result of the use of immunosuppressive medications.

### Impact of co-existing PSC on disease behavior and course of UC after colectomy

Patients with PSC-UC are also at an increased risk of pouchitis after restorative proctocolectomy [38, 39, 43]. On the other hand, the risk for pouchitis does not appear to be related to the severity of PSC [43]. Concurrent PSC appears to be associated with a significant pre-pouch ileitis on endoscopy and histology in patients with IPAA. Pouch patients with a long segment of ileitis should be evaluated for PSC. The presence of PSC is inversely associated with the development of CD of the pouch [60].

### Impact of co-existing UC (before colectomy) on disease behavior and course of PSC before liver transplantation

The presence of IBD may affect the disease course and behavior of PSC. An early study comparing PSC patients with and without UC showed no difference on liver histology [61]. Two subsequent studies showed lack of specific distinguishing clinical and radiographical features of PSC-IBD from PSC without IBD [62, 63]; however, another study suggested that certain clinical, laboratory, and radiographical features might be useful in distinguishing patients with and without co-existing IBD [64].

In our center's study, PSC was often recognized at an early stage in patients with concurrent UC [65]; UC however did not impact on long-term prognosis in terms of liver-related outcomes when adjusted for the severity of liver disease [66]; however, in a population-based study, concurrent IBD influenced age at diagnosis, development of cancer, mortality, and requirement for OLT in patients with PSC [66].

### Impact of co-existing UC (after colectomy) on disease behavior and course of PSC after liver transplantation

**Figure 3.**
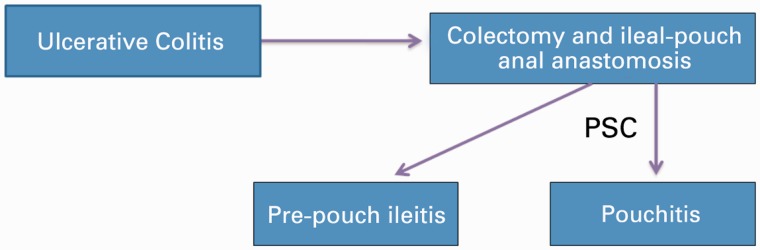
The figure illustrates the relationship between primary sclerosing cholangitis (PSC) and ulcerative colitis (UC) after colectomy. There is higher risk of pre-pouch ileitis and pouchitis after colectomy.

The relationship between restorative proctocolectomy with IPAA and the course of PSC has been evaluated. IPAA does not appear to affect the disease course of PSC and PSC appears to follow an independent disease course despite proctocolectomy [67–70]. Chronic antibiotic refractory pouchitis (CARP) is common in patients with ulcerative colitis and PSC. OLT in these patients may not affect the frequency of CARP in general and appears not to alter the disease course of pre-existing CARP; however, in a subset of patients, OLT might reduce the risk for the development of *de novo* CARP [71]. PSC-associated IBD patients, after restorative proctocolectomy, have a higher risk of neoplasia/cancer in the resected specimen, post-operative pelvic sepsis, and higher long-term mortality [68, 70]. Quality of life remains similar in IBD patients after restorative proctocolectomy, with or without associated PSC in the follow-up Figure 3.

The presence of the IPAA in OLT for PSC patients appears not to have a negative impact on patient- and graft survivals and post-operative complications in liver transplants [72].

### Impact of UC activity on PSC recurrence after liver transplantation

The presence of active inflammation in the colon may increase the risk of recurrence of PSC in the allograft. A previous study showed that use of corticosteroids to treat IBD flares following OLT may be associated with recurrence of PSC in the allograft [73]. Aggressive measures to prevent colon inflammation in the post-OLT setting may be required to protect the liver from recurrence of PSC.

### Natural history

The natural history of PSC varies. The Composite Mayo Risk Score has been used to assess disease progression and prognosis [74].

### Cancer risk and impact of liver–gut crosstalk

Annual incidence of cholangiocarcinoma (CCA) was reported in 0.6–1% of PSC patients [75]. CCA can arise at any stage of PSC and up to 50% of CCA is diagnosed within the first year following diagnosis [76]. Endoscopic retrograde pancreatocholangiography (ERCP) and brush cytology of strictures is the first step in the diagnosis of CCA [77]; however, brushings for both cytology and Fluorescence in situ hybridization are limited by poor sensitivity in diagnosis of CCA. These patients are also at increased risk for gall bladder cancer; however, there is so far no consensus as to what should trigger cholecystectomy in these patients. In addition to CCA and gall bladder cancer, PSC patients are also at an increased risk for pancreatic cancer, compared with the general population [74, 78].

In a recent meta-analysis, brushings for both cytology and FISH are less sensitive for diagnosis of CCA [79, 80]. The technique of brushing is probably also of importance, but has not been studied systematically. Experts in Scandinavia have obtained high-quality samples by sampling non-dilated strictures, using an over-the-wire brush [81]. They have suggested that brushing with the whole catheter—as opposed to moving the brush in and out of the catheter—may increase the yield, avoiding loss of material against the edge of the catheter [82]. Development of lipidomics-based assay could improve the diagnosis of CCA in these patients [83].

The relationship of liver–gut crosstalk to CCA is not clear. It is usually associated with PSC seen in patients with IBD; an earlier study reported an association between IBD and CCA [84]. The incidence and survival of CCA in IBD patients was also studied in a national database review from Denmark [85]. The incidence of CCA among the 41 280 IBD patients was increased four-fold, compared with controls. The 10-year cumulative risk of cholangiocarcinoma in IBD patients was 0.07%. The risk was much higher in males and in patients with UC. CCA patients with IBD were, on average, 15 years younger at cancer diagnosis than those who were IBD-free [85]. In a population-based study from Manitoba, an increased incidence of hepatobiliary cancers was observed, both in CD patients (5.22; 95% CI: 0.96–28.5) and in UC patients (3.96; 95% CI: 1.05–14.9) [86].

However, recent studies of CCA in PSC patients did not identify IBD as a risk factor [87, 88]. In a recent Dutch study, age at presentation of PSC was identified as the only independent risk factor; IBD was not identified as a risk factor [89]. Also in a study of 100 consecutive formalin-fixed PSC liver explants (including 30 with CCA), there were no significant differences in age, sex, history of IBD, or PSC duration on risk of cholangiocarcinoma. Even in the absence of cancer, bile duct dysplasia was seen, at least focally, in 36% of benign end-stage PSC explants [90].

### Management

The treatment strategy for PSC, with or without IBD, is similar. None of the currently used agents has shown convincing benefits in preventing the progression of PSC or altering the natural history of the disease. Ursodeoxycholic acid (UDCA) has been used extensively [91, 92]; while a pilot study suggested that even a higher dose of UDCA (28–30 mg/kg/day) might improve survival [93], a large, multicenter, randomized trial was halted early due to a significant increase in adverse events in the study group [94]. In a retrospective analysis of 139 PSC patients, those receiving UDCA and achieving an improvement of Alkaline Phosphatase (ALP) to <1.5 x ULN had significantly longer survival, without end points, compared with patients without ALP normalization [95].

Antibiotics are currently being explored for treatment of PSC [96–98]. Non-absorbable antibiotics, including vancomycin, have been shown to influence regulatory T-cell subsets. In a pediatric study, liver tests improved with the use of vancomycin and this was associated with increased levels of regulatory T-cells [96]. As further research develops, seeking to understand the genomics and gut microbiome in PSC pathogenesis, the use of antibiotics or pro-biotics may play a greater role.

Endoscopic intervention against PSC is often needed in patients with rapidly progressive disease, worsening jaundice, cholangitis, or suspicion of cholangiocarcinoma, to evaluate for dominant strictures. In PSC patients with dominant strictures, endoscopic balloon dilation of strictures is performed, with or without stent placement. A multicenter, prospective, randomized trial to compare the efficacy of balloon dilatation against dilation with short-term stenting is currently in progress.

OLT is the treatment of choice for end-stage PSC or PSC with CCA. The outcome of OLT in PSC is relatively favorable with 5- and 10-year survival rates of 85% and 70%, respectively [99].

## AUTO-IMMUNE HEPATITIS/PSC OVERLAP SYNDROME

AIH/PSC overlap has been described in patients with IBD, particularly those with UC [100]. The diagnosis is suspected when there is a definite diagnosis of AIH based on the International Autoimmune Hepatitis Group criteria with a total score >15 points [101]. Immunosuppressive therapy for treatment of AIH, along standard guidelines, is recommended for PSC patients with features overlapping AIH.

## IgG4-ASSOCIATED CHOLANGITIS

IgG4-associated cholangitis (IAC) has been described in patients with auto-immune pancreatitis (AI), being a part of IgG4-related systemic disease or being a separate disease entity [102, 103]. Increased serum IgG4 level is also observed in 9% of patients with PSC [104]. Increased numbers of IgG4+ plasma cells on histology were found in 23% of explanted liver specimens from 99 patients with OLT [105]. IAC was described in two patients with concurrent UC [106]. Although slight elevations of serum IgG4 occur in PSC patients who do not fulfill the criteria for IAC, these patients experience a more aggressive disease course, with shorter time to OLT. In PSC patients who have elevated IgG4, it has also been demonstrated that these patients are more likely to have backwash ileitis and had shorter colectomy-free survival than patients with normal IgG4 [53]; hence, evaluation of serum IgG4 needs to part of clinical evaluation in PSC patients.

## CONCLUSIONS

Our review illustrates the close liver–gut crosstalk in the pathogenesis of PSC and IAC with gut inflammation in IBD. Ongoing research in metagenomics will better understand the relationship of liver–gut crosstalk to pathogenesis and provide us with management options for these challenging PSC patients.

**Conflict of interest:** none declared.
